# Enabling Complex Queries to Drug Information Sources through Functional Composition

**Published:** 2013

**Authors:** Lee Peters, Jonathan Mortensen, Thang Nguyen, Olivier Bodenreider

**Affiliations:** a National Library of Medicine, National Institutes of Health, Bethesda, Maryland, USA; b Stanford Center for Biomedical Informatics Research, Stanford University, Stanford, California, USA 94305

**Keywords:** RxNorm, NDF-RT, application programming interface, web service composition, complex queries

## Abstract

Our objective was to enable an end-user to create complex queries to drug information sources through functional composition, by creating sequences of functions from application program interfaces (API) to drug terminologies. The development of a functional composition model seeks to link functions from two distinct APIs. An ontology was developed using Protégé to model the functions of the RxNorm and NDF-RT APIs by describing the semantics of their input and output. A set of rules were developed to define the interoperable conditions for functional composition. The operational definition of interoperability between function pairs is established by executing the rules on the ontology. We illustrate that the functional composition model supports common use cases, including checking interactions for RxNorm drugs and deploying allergy lists defined in reference to drug properties in NDF-RT. This model supports the RxMix application (http://mor.nlm.nih.gov/RxMix/), an application we developed for enabling complex queries to the RxNorm and NDF-RT APIs.

## Introduction

Biomedical terminologies are important knowledge sources for many aspects of biomedical research and healthcare [1, 2]. In particular, standard terminologies, such as SNOMED CT, LOINC, and RxNorm, play a crucial role in health information exchange and the certification of electronic health record technology, commonly referred to as “meaningful use” [3, 4]. Access to biomedical terminologies is either direct, interactive access by a user through a browser, or access by a software application through an application programming interface (API).

Many browsers have been developed to access biomedical terminologies. For example, there are over twenty browsers for SNOMED CT [5]. Biomedical terminologies can also be accessed through repositories, such as the National Library of Medicine’s Unified Medical Language System (UMLS) [6] and the National Center for Biomedical Ontology’s BioPortal [7]. The UMLS and BioPortal both offer web-based browsers through which users can find codes for a given biomedical term, navigate hierarchical and other relations, and explore mappings across terminologies.

In order to support access to biomedical terminologies by software applications, application programming interfaces (APIs) have been developed, often based on web services. These APIs are a key component of health information technologies, including “meaningful use”, as they mediate access to standard terminologies through transport standards, such as the Simple Object Access Protocol (SOAP) and REpresentational State Transfer (REST) architecture. Examples of APIs to biomedical terminologies include the UMLS Terminology Services API (SOAP-based) [8], the BioPortal API (RESTful) [9], and the APIs to drug information sources developed for RxNav (SOAP-based and RESTful) [10]. APIs to biomedical terminologies have been developed independently of one another and are generally poorly interoperable. Standardization of terminology services by standard development organizations, such as the Object Management Group (OMG) and Health Level 7 (HL7) is underway through the specification of Common Terminology Services 2.0 (CTS2) [11].

Terminology APIs generally offer a set of basic functions that can be used and combined by a user to obtain relevant terminological information. Typical functions include finding the code associated with a string, accessing the properties of a concept, getting the list of related concepts for a given relationship, and getting the list of codes in a given terminology. API developers do not normally offer functions for complex queries because they rarely know in advance all the use cases for the API. While simple functions offer the best chances for reuse, composing complex queries remains challenging for users, because it requires higher programming skills than access to simple functions, and frameworks for composing web services are not available for most APIs.

The objective of this work is to enable an end-user to create complex queries to drug information sources through functional composition. In practice, we propose to allow users to specify and execute a sequence of web service functions. In our typical scenario, users select functions from two different web services to drug information sources – RxNorm API and NDF-RT API, and specify a “workflow” of operations to execute in sequence. An ontology, which specifies web service function interoperability, facilitates the workflow creation process in our application.

For example, suppose an application needed to find all the brand name products available for a given generic drug, whose identity is known by the FDA unique ingredient identifier code (UNII_CODE). To do this using the RxNorm API^4^, the following steps would be performed (SOAP API functions listed in parenthesis).

1. Translate the UNII_CODE into an RxNorm identifier (findRxcuiById).
2. Find the related branded drugs (getRelatedByType).

The result returned in the first step is an RxNorm concept identifier (RxCUI), which is used in the second step as a calling argument to the API function getRelatedByType. This function returns related RxNorm concepts from the RxCUI. By enabling functional composition, we make this example workflow readily available to end-users.

## Background

### Web service composition

We considered a number of existing web service annotation and composition frameworks to help define the function composition model which was introduced above.

- Semantic Annotations for WSDL and XML Schema (SAWSDL) is a technical recommendation published by the World Wide Web Consortium (W3C) in 2007 in the context of Semantic Web Framework [12]. The specification enables semantic annotations for Web services using and building on the existing extensibility framework of WSDL. However, SAWSDL has not gained wide use, nor does it provide a means for composition.
- SSWAP (Simple Semantic Web Architecture and Protocol) aims to combine web services and semantic web technologies to enable high-throughput discovery, assessment, and integration of data and services between distributed parties [13]. Semantic Web ontologies encoded in OWL are used to describe information about a web service such as the service category, types of input the service consumes, and the types of output the service produces. Retrofitting our web services to meet SSWAP compliance was not considered feasible. So far, SSWAP has only been adopted by a small number of bioinformatics resources.
- The Semantic Automated Discovery and Integration (SADI) is a set of standards-compliant best practices that simplify interoperability between semantic web services [14]. Using Semantic Web technologies, SADI services consume and produce OWL classes. While SADI is directly compatible with web services standards, it is best suited to the development of new web services, for which it provides guidelines. Retrofitting our web services to meet SADI compliance was not an option in our case.
- Finally, the workflow application Taverna [15] does not provide any type of semantic validation.

We chose to develop an OWL ontology that describes the web services we have developed for the RxNorm and NDF-RT drug information sources. As with SSWAP and SADI, this ontology describes the semantics of the input and output of each function. Unlike other frameworks, however, this ontology is only used as background knowledge for our web services composition application, not in the payload of our web services, which remains unchanged.

### Drug information sources

**RxNorm** is a standardized nomenclature for medications produced and maintained by the U.S. National Library of Medicine (NLM) in cooperation with proprietary vendors [16]. RxNorm concepts are linked by NLM to multiple drug identifiers for each of the commercially available drug databases within the UMLS^®^ Metathesaurus^®^. In addition to integrating names from existing drug vocabularies, RxNorm creates standard names for clinical drugs. The **RxNorm API** provides functionality to access the RxNorm data set, including mapping from identifiers of other drug vocabularies and identification of clinical and branded drug concepts through a set of named relationships [10]. The SOAP version of the API contains 28 functions with equivalent functionality in a RESTful API implementation.

**National Drug File Reference Terminology (NDF-RT)** is a concept oriented terminology whose concepts are organized into taxonomies [17]. In NDF-RT™, generic ingredients or combinations thereof are described in terms of their active ingredients, mechanisms of action, physiologic effects, and therapeutics (indications and contraindications). Orderable (clinical) drug products inherit the descriptions of their generic ingredients, and are further described by local (VHA) drug classification, strength, units, and dose forms. The **NDF-RT API** contains functionality to access the hierarchy of data associated with ingredients and clinical drugs [10].

## Methods

### Principles for web service composition

Our primary focus in developing a web service composition model is to accurately determine the interoperability between functions. For two functions to be interoperable, one function must produce as output an element or structure that semantically matches the input needed by another function. By semantically representing the function inputs and outputs, and then applying a set of matching rules to the representations, the interoperability between functions can be discovered.

### Modeling web services functions in the ontology

To model the web services functions, we developed an ontology using Protégé. The main focus of the ontology is the description of functions in a web service. The following tables describe the components (Table 1) and properties (Table 2) of the ontology.

### Modeling web services composition through rules

To supplement the ontology, we developed a set of rules to determine the semantic interoperability of the functions for web service composition as presented earlier. The rules are listed in Table 3. Note: interoperability is one-directional.

### Instantiating the model and inferring interoperability relations

Web services are modeled semantically utilizing the framework provided above. In the ontology, the model is described using classes. The specific function parameters are instances of the classes. The properties provide the relationships between classes and are represented as triples. In our model for example:

“RxNorm API” “has_function” “findRxcuiByID”

“findRxcuiById” “has_input” “id_type”

“findRxcuiById” “has_input” “id”

“findRxcuiById” “has_output” “RxCUI”

“RxNorm API” “has_function” “getRelatedByType”

“getRelatedByType” “has_input” “RxCUI”

The above example provides a representation of the fact that the RxNorm API contains the functions findRxcuiById and getRelatedByType, and describes the inputs and outputs of findRxcuiById and an input of getRelatedByType.

Once all the functions and the properties are specified in the ontology, then the rules are applied to generate a set of inferred relations (triples). The inferred relations include the identification of the interoperability between two functions. In our example above, the following triple is generated:

“findRxcuiById” “interoperable_with” “getRelatedByType”

The triples of the ontology are stored in a Virtuoso [19] database, and a set of API functions was developed to access this data, including one function to extract all the interoperability relations.

## Use Cases

Our web service composition model supports a number of common use cases. Most use cases involve the use of more than one API but complex queries within one API are also possible. Several use cases suggested by our users are listed below. Up until now, implementation of the use cases required *ad hoc* programming for web service composition, and was a hindrance to the use of complex queries.

### Finding clinical drugs which may cause allergic reactions

In this use case, a user is interested in finding all the clinical drugs known in RxNorm that contain an ingredient class (example: penicillins) which a patient might be allergic to. A workflow can be constructed from the API functions in NDF-RT and RxNorm APIs.

1. findConceptsByName from NDF-RT API to identify the ingredient class
2. findChildConcepts from NDF-RT API to identify all the children of the ingredient class
3. getRelatedConceptsByReverseRole from NDF-RT API, specifying “has_ingredient” as the role to identify the drug level concepts
4. findRxcuiById from RxNorm API to identify the RxNorm concept for the ingredient
5. getRelatedByType from RxNorm API, specifying “SCD” as the term type, to identify the clinical drugs associated with the ingredient.

Example: Find the clinical drugs containing hydantoins (the allergic condition). The output of this workflow is a list of 49 clinical drugs from RxNorm, including drugs containing allantoin, dantrolene, ethotoin, fosphenytoin, mephenytoin, and phenytoin (for example “Phenytoin 30 MG Oral Capsule”).

### Finding interactions to clinical drugs

A user wishes to update the list of drug interactions to clinical drugs specified by RxCUIs. Since the list of clinical drugs is old, a check needs to be made to see if these drugs are still active or have been remapped into new concepts in RxNorm. The workflow of functions would use both the RxNorm and NDF-RT APIs.

1. getRxcuiStatus from RxNorm API to determine if the concept is still active or has been remapped
2. getRelatedByType from RxNorm API to get the ingredients in the clinical drug
3. findConceptsById from NDF-RT API to get the NDF-RT identifiers for the ingredients
4. findDrugInteractions from NDF-RT API to get the ingredients that interact with the clinical drug ingredients.

Example: Find the interactions to a sulfamethoxazole 800mg – trimethoprim 160 mg oral tablet (RxCUI = 198335). The output from the workflow is a list of interactions containing 14 drugs for sulfamethoxazole (for example Dicumarol), and 11 drugs for trimethoprim (for example Warfarin).

### Finding ingredients from clinical drugs

One user needs to determine the NDF-RT ingredient identifier starting from a clinical drug identified by an RxCUI. The following operations are performed:

1. A call to the RxNorm API function getRelatedByType to get the corresponding ingredient concept(s) related to the clinical drug
2. A call to the NDF-RT API function findConceptsById to map the RxNorm ingredient concepts to NDF-RT concepts.
3. A call to the NDF-RT API function getConceptProperties to find those concepts that were designated as ingredients.

Example: Find the NDF-RT ingredients starting with RxCUI = 860232. The output of this workflow is the ingredient concepts in NDF-RT for Guaifenesin, Phenylephrine, and Hydrocodone.

### Finding VA classes for clinical dose forms

Another use case is finding the VA classes for clinical dose forms. For example: What is the VA class for clofazimine oral tablets (RxCUI=371567)? A workflow can be constructed using a web service composition application to answer this question.

1. getRelatedByType from RxNorm API, specifying “SCD” as the term type.
2. findConceptsById from NDF-RT API, specifying “RXCUI” as the Id type.
3. getVaClassOfConcept from NDF-RT API.

Example: Find the VA class for clofazimine oral tablets (RxCUI = 371567). The output of this workflow is the VA class “Anti-Infectives, Other”.

### Finding brand names from clinical drug strings

MedlinePlus Connect [18] uses the RxNorm API to find brand names associated with clinical drug name strings. A simple workflow can be constructed to accomplish this.

1. findRxcuiByName from RxNorm API, specifying normalized string search
2. getAllRelated from RxNorm API, to get the related brand information.

Example: Find the brand information for the name “citalopram 20 mg tablet”. The output of this workflow will return the brand information for Celexa.

## Discussion

### Significance

This work is not merely an incremental improvement over the RxNorm and NDF-RT APIs we have developed in the past years. From a clinical perspective, it is driven by common use cases for which complex queries involving multiple API function calls are required. In our experience, it is difficult for most users to generate such queries. By guiding users in the composition process, the web service composition model facilitates creating such queries. From a technical perspective, the fact that the interoperability ontology resides outside the software of the web services itself allows for easy maintenance of both the software and the ontology.

### Maintenance

The web service composition model is easily expanded to add a new function of a web service. This is done by adding the description of the function (primarily the inputs and outputs) and executing the rule set to generate the interoperability with the other functions.

For example, to add the NDF-RT API function getVAClassMembers, the following steps are performed.

1. The instance of the function class is defined with the value “getVAClassMembers”.
2. The input for the function is defined: the property “has_input” has a value of “NUI”
3. The output is defined: the property “has_output” would have a value of “minimal_concept”. Note that “minimal_concept” has been previously defined and “has_members” of “term_type”, “RxCui” and “Name”.
4. The rules are applied and new operability pairings are generated. In this case, any function which produces a NUI as output would be interoperable with getVAClassMembers. For example, “getChildConcepts” is “interoperable_with” “getVAClassMembers”. Similarly, since getVAClassMembers produces RxCui and a concept name as output, so any function that receives either of those as input will be potentially interoperable. For example, “getVAClassMembers” is “interoperable_with” “findDrugInteractions”.

### Limitations

The web service composition model is not compliant with broader frameworks like SADI. Because the APIs were well established with a large client base, we made a conscious decision not to change them to conform to those frameworks. In future work we are planning to investigate how our framework could be made compatible with SADI.

The model produces *possible* interoperable function pairs, but these may not be *practical* pairings. The application using the interoperable data may need to eliminate some of these pairings for many different reasons.

### Application

**Figure 1:**
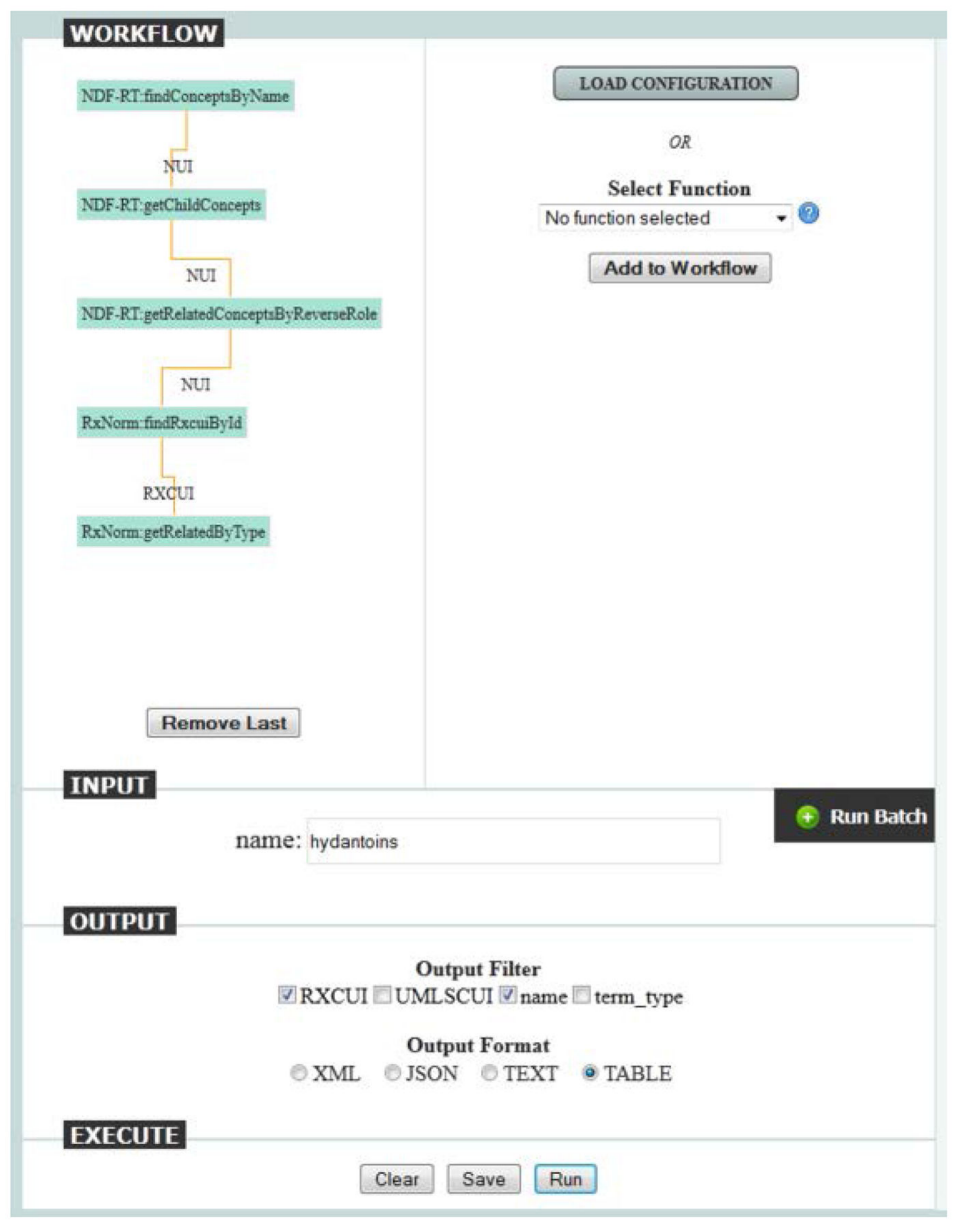
Example of web service composition workflow in the RxMix application.

We have developed and recently released RxMix (http://mor.nlm.nih.gov/RxMix/), a web service composition application for enabling complex queries to the RxNorm, RxTerms and NDF-RT APIs. This application allows biomedical researchers and health professionals to interactively create complex workflows (i.e. sequences of interoperable API functions) through a graphical user interface,, without having to write programs. Workflow creation and validation is supported effectively by the web service composition model (and ontology) we have developed. Once created, these workflows can be executed on lists of entities (e.g., find brand names for a list of NDC codes). Figure 1 shows the workflow for the “allergy” use cases.

## Conclusions

We proposed a web service composition model for the RxNorm and NDF-RT APIs. This model enables an end-user to create complex queries to drug information sources through functional composition, by creating sequences of functions from application program interfaces (API) to these drug terminologies. We illustrate that the functional composition model supports common use cases, including checking interactions for RxNorm drugs and deploying allergy lists defined in reference to drug properties in NDF-RT.

## Figures and Tables

**Table 1:** List of components in the interoperability ontology.

Components/classes	Description
service	group of functions, may have a set of sources and specific IDs. Example: RxNorm API
function	a specific function of a service, has input and output parameters. Example: findRxcuiById
parameter	semantically described characteristics of input and output of a function. Example: RxCUI
source	certain vocabularies a service may have. Example: RxNorm
workflow_element	a container to describe a unit in a workflow, each containing a function and set of data annotations

**Table 2:** List of properties in the interoperability ontology.

Properties	Description
has_function	relates a service to a function
has_id	relates a source to an ID
has_initial_output	relates the first workflow element (the user input) to the annotation of the input
has_input	relates a function to expected inputs or relates a workflow element to actual data inputs
has_member	relates a user_defined parameter to other parameters
has_output	relates a function to an output parameter
has_source	relates a service to a source
interoperable_with	relates a function to another function
next_element	points a workflow element to the next workflow element
previous_element	points a workflow element to the previous workflow element
provided_by	relates a function to a service
provides	relates a service to a function

**Table 3:** List of rules defining interoperability among functions.

**Rules** – Given: a function *A* might be interoperable with function *B*, potentially across APIs (services)
If the input of *B* matches the output of *A*, then *A* is potentially interoperable with *B*
If *A* has an output composed of members (a non-primitive output), then *A* also has as output those members (transitively)
If the output of *A* is a general ID, the input of *B* is specific ID, and the set of potential IDs for *A* (inferred through the sources of *A*’s service) contain the specific ID of *B*, then *A* is potentially interoperable with *B*
If the output of *A* is a specific ID and the input to *B* is a general ID, and the potential IDs for *B* (inferred through the sources of *A*’s service) contain the specific ID of *A*, then *A* is potentially interoperable with *B*

## References

[1] Bodenreider O (2008). Biomedical ontologies in action: role in knowledge management, data integration and decision support. Yearb Med Inform.

[2] Cimino JJ, Zhu X (2006). The practical impact of ontologies on biomedical informatics. Yearb Med Inform.

[3] Blumenthal D, Tavenner M (2010). The “meaningful use” regulation for electronic health records. N Engl J Med.

[4] Health and Human Services Department (2012). Health Information Technology: Standards, Implementation Specifications, and Certification Criteria for Electronic Health Record Technology, 2014 Edition; Revisions to the Permanent Certification Program for Health Information Technology: A Proposed Rule by the Health and Human Services Department on 03/07/2012. Federal Register.

[5] Rogers J, Bodenreider O (2008). SNOMED CT: Browsing the browsers. Proceedings of the Third International Conference on Knowledge Representation in Medicine (KR-MED.

[6] Bodenreider O (2004). The Unified Medical Language System (UMLS): Integrating biomedical terminology. Nucleic Acids Res.

[7] Whetzel PL, Noy NF, Shah NH, Alexander PR, Nyulas C, Tudorache T (2011). BioPortal: enhanced functionality via new Web services from the National Center for Biomedical Ontology to access and use ontologies in software applications. Nucleic Acids Res.

[8] National Library of Medicine UMLS Terminology Services.

[9] National Center for Biomedical Ontology BioPortal.

[10] National Library of Medicine RxNav.

[11] Object Management Group (2011). CTS2.

[12] World Wide Web Consortium Semantic Annotations for WSDL and XML Schema.

[13] University of Arizona Simple Semantic Web Architecture and Protocol.

[14] Wilkinson MD, Vandervalk B, McCarthy L (2011). The Semantic Automated Discovery and Integration (SADI) Web service Design-Pattern, API and Reference Implementation. J Biomed Semantics.

[15] Hull D, Wolstencroft K, Stevens R, Goble C, Pocock MR, Li P (2006). Taverna: a tool for building and running workflows of services. Nucleic Acids Res.

[16] Nelson SJ, Zeng K, Kilbourne J, Powell T, Moore R (2011). Normalized names for clinical drugs: RxNorm at 6 years. J Am Med Inform Assoc.

[17] Veterans Health Administration (2010). NDF-RT.

[18] National Library of Medicine MedlinePlusConnect.

[19] Virtuoso http://docs.openlinksw.com/virtuoso/.

